# Medical Students and Graduates in the United States during the Session of 1860–61

**Published:** 1861-05

**Authors:** 


MEDICAL STUDENTS AND GRADUATES IN THE UNITED STATES DURING THE SESSION OF 1860-61.—From the following list of the number of students in attendance at the different medical institutions of this country, it will be perceived that the classes of the last session were, in the aggregate, not so large as those of the previous one. Although all our schools are not represented, simply from our not having received catalogues, the list is, so far as we can learn, correct.

| | *Students.* | *Graduates.* |
|---|---|---|
| Jefferson Medical College | 443 | 187 |
| University of Pennsylvania | 465 | 175 |
| Pennsylvania Medical College | | 38 |
| University of New York | 420 | 129 |
| College of Physicians and Surgeons | 264 | 62 |
| New York Medical College | | 17 |
| University of Maryland | 150 | 63 |
| Medical College of South Carolina | 222 | 93 |
| Massachusetts Medical College | 205 | 39 |
| New Orleans School of Medicine | 236 | 76 |
| University of Louisiana | 401 | 132 |
| St. Louis Medical College | 140 | 52 |
| Medical College of Ohio | | 24 |
| Cincinnati College of Medicine and Surgery | 127 | 36 |
| Starling Medical College | | 16 |
| University of Nashville | | 141 |
| Memphis Medical College | | 21 |
| Long Island College Hospital | 58 | 21 |
| National Medical College | 83 | |
| Medical College of Virginia | | 59 |
| Savannah Medical College | | 14 |
| Alabama Medical College | | 31 |
| Lind University | 51 | 12 |
| Rush Medical College | | 37 |
| University of Michigan | 242 | 21 |

